# Structure of Plasma (re)Polymerized Polylactic Acid Films Fabricated by Plasma-Assisted Vapour Thermal Deposition

**DOI:** 10.3390/ma14020459

**Published:** 2021-01-19

**Authors:** Zdeněk Krtouš, Lenka Hanyková, Ivan Krakovský, Daniil Nikitin, Pavel Pleskunov, Ondřej Kylián, Jana Sedlaříková, Jaroslav Kousal

**Affiliations:** 1 Faculty of Mathematics and Physics, Charles University, V Holešovickách 2, 180 00 Prague, Czech Republic; krtousz@gmail.com (Z.K.); hanykova@kmf.troja.mff.cuni.cz (L.H.); ivank@kmf.troja.mff.cuni.cz (I.K.); daniilnikitin92@gmail.com (D.N.); pleskunov@protonmail.ch (P.P.); ondrej.kylian@gmail.com (O.K.); 2 Faculty of Technology, Tomas Bata University in Zlín, Vavrečkova 275, 76001 Zlín, Czech Republic; sedlarikova@utb.cz; 3 Centre of Polymer Systems, Tomas Bata University in Zlín, Třída Tomáše Bati 5678, 76001 Zlín, Czech Republic

**Keywords:** plasma polymerisation, plasma-assisted vapour thermal deposition, thin films, XPS analysis, NMR analysis

## Abstract

Plasma polymer films typically consist of very short fragments of the precursor molecules. That rather limits the applicability of most plasma polymerisation/plasma-enhanced chemical vapour deposition (PECVD) processes in cases where retention of longer molecular structures is desirable. Plasma-assisted vapour thermal deposition (PAVTD) circumvents this limitation by using a classical bulk polymer as a high molecular weight “precursor”. As a model polymer in this study, polylactic acid (PLA) has been used. The resulting PLA-like films were characterised mostly by X-ray photoelectron spectroscopy (XPS) and nuclear magnetic resonance (NMR) spectroscopy. The molecular structure of the films was found to be tunable in a broad range: from the structures very similar to bulk PLA polymer to structures that are more typical for films prepared using PECVD. In all cases, PLA-like groups are at least partially preserved. A simplified model of the PAVTD process chemistry was proposed and found to describe well the observed composition of the films. The structure of the PLA-like films demonstrates the ability of plasma-assisted vapour thermal deposition to bridge the typical gap between the classical and plasma polymers.

## 1. Introduction

Plasma polymers are macromolecular solids created when an organic vapour or precursor passes through a glow discharge. In contrast to conventional polymers, plasma polymers have random, highly branched structure and typically exhibit a much higher degree of crosslinking. Although such materials were observed in the 19th century (see for instance [1,2] and references therein) they were, due to the lack of predictable structure, considered as nuisance by-products of plasma processing for a long time. This situation changed at the beginning of the 1960s when it was shown that plasma polymerised styrene might be used as a dielectric separation membrane for nuclear battery [3]. Since then, the range of possible applications of plasma polymers rapidly increased, and plasma polymers are nowadays applied in such diverse areas as microelectronics, automotive, food packaging, textile industries, or in the biomedical field. They can act as separation, protective or barrier layers [4,5,6,7,8] or as films that modify the wettability or biofouling of coated objects [9,10,11,12,13,14,15,16,17].

The massive spread and ongoing popularity of plasma polymerisation are connected with the unique features of this technique. These refer to its dry and solvent-free character, the possibility to polymerise monomers that do not polymerise by conventional chemical routes, which allows for the production of materials with properties not achievable by wet chemistry, as well as to the possibility to deposit thin and conformal films on virtually any substrate material. Naturally, these features triggered-off intensive research of plasma polymers intending to produce materials with tailor-made properties. As shown in numerous studies, the structure of plasma polymers, along with their functional properties (e.g., bioadhesivity, wettability, elastic modulus, roughness, solubility or permeability), may be controlled in a wide range by the deposition conditions. However, as only easily vaporised precursors/monomers may be used in the conventionally used PECVD, the plasma polymerisation is limited to low-molar-mass volatile substances. This, in turn, restrains the attainable structures of plasma polymers synthesised by the PECVD technique. To overcome this general limitation, plasma-assisted vapour (vacuum) thermal deposition (decomposition/degradation) (PAVTD) was introduced as an alternative route of production of plasma polymers [18,19,20,21,22,23]. In this technique, the polymer is heated at reduced pressure in an inert atmosphere to a temperature at which the thermal decomposition of its macromolecular chains occurs. This generates a flux of both low- and high-molar mass species that are subsequently activated or fragmented by the plasma that initiates the polymerisation process. As was shown, e.g., by analyses of molar mass, swelling and composition, the final structure of such produced coatings may be tuned simply by the applied power from highly crosslinked one for high applied powers to the structure with partial preservation of the polymer chains present in the initial polymer at low powers [19,21,23,24,25]. In other words, this technique allows for the filling of the gap between the fully aperiodic plasma polymers synthesised by PECVD and polymers prepared by classic wet chemistry and thus to widen further the range of achievable functional properties of plasma polymers.

Among different types of polymer, an important class constitute bioplastics, i.e., plastics that can be produced from renewable sources. Polylactic acid is a typical example of such materials that is, due to its biocompatibility and biodegradability proceeding by the hydrolysis of ester bonds, considered as a highly valuable material for short-term food packaging, production of scaffolds for tissue engineering or as a drug carrier material [26,27,28,29,30]. In some of the aforementioned applications, it is, however, crucial not only to tailor the degradation rate but also to produce PLA-based materials in the form of thin pin-hole free films with well-controlled thickness. As already mentioned, the latter two requirements may be easily fulfilled by plasma polymerisation, and thus the possibility to produce PLA-like plasma polymers by plasma-based methods receives increasing attention. For instance, the PLA-like thin films were already produced by plasma polymerisation of ethyl lactate performed both at reduced [31,32] or atmospheric pressure [33,34]. However, the relatively low retention of PLA-character was reported for such produced plasma polymer coatings that were characterised by O/C ratios lower than ~0.35, i.e., the value almost two times lower as compared to the original PLA, for which the O/C ratio is 0.66. More recently, the preliminary study indicated the possibility to increase the PLA character of prepared plasma polymer films when PAVTD is applied [25], however, no systematic study of the relation between the deposition parameters and chemical structure of fabricated PLA-like coatings was performed. Due to this fact, the main aim of this investigation was to provide a detailed characterisation of PAVTD deposition of PLA-like plasma polymers in dependence on the applied power. To meet this general aim, PAVTD of PLA was performed at applied powers up to 120 W, and the produced coatings were thoroughly analysed from the point of their structure by a combination of X-ray photoelectron spectroscopy (XPS) and nuclear magnetic resonance (NMR). Based on this analysis, a model of growth of PAVTD-deposited plasma polymers was suggested also.

## 2. Materials and Methods

### 2.1. Deposition of Plasma Polymerised Polylactic Acid (PLA) Coatings

**Figure 1:**
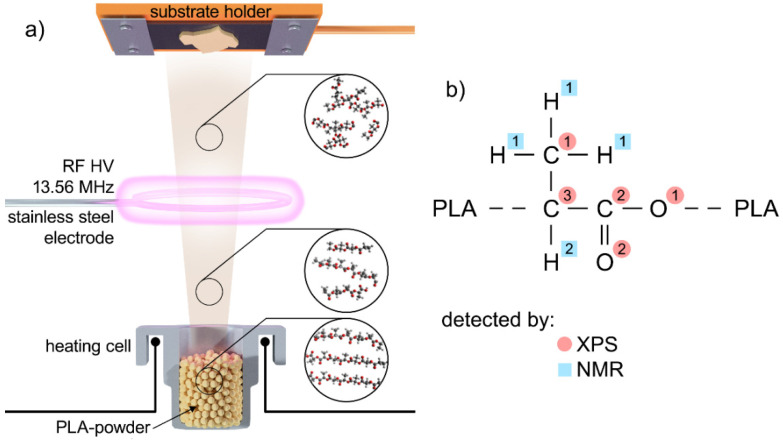
(**a**) Experimental set-up used for PAVTD of PLA-like coatings and (**b**) chemical structure of conventional PLA with the assignment of C, O and H that corresponds to different spectral peaks detectable by XP) and NMR, respectively.

The deposition system, schematically depicted in Figure 1a, consisted of a stainless steel high vacuum chamber (volume 35 L), equipped with an electrically heated copper crucible loaded with PLA powder (prepared according to [35], molar mass M_n_ = 8300 g/mol; M_w_ = 19,000 g/mol, the chemical structure of which is presented in Figure 1b). Above the crucible, a circular excitation electrode was placed at the distance of 4 cm, and quartz crystal microbalance sensor (QCM) was placed at a distance of 10 cm. The load-lock system with the sample holder was placed 15 cm above the crucible. The following procedure was employed for the production of the samples. First, the vacuum chamber was pumped (~1 h) down to the base pressure of 1 × 10^−3^ Pa by rotary (5 m^3^/h) and diffusion (250 L/s) pumps. Subsequently, Ar gas was introduced into the deposition chamber through a flow controller (MF1, MKS, Andover, MA, USA) at the flow of 7 cm^3^/min_STP_ (STP: at standard pressure), and the pressure of 5 Pa. As soon as the temperature (~220 °C) at which a flux of evaporated species (decomposed fragments of the PLA macromolecules) is generated was reached, the power (0–120 W) was delivered to the antenna via a matching unit from a radio frequency (RF) generator (Elite 300, 13.56 MHz, 300 W, MKS, Andover, MA, USA). When the deposition monitored by the QCM had been adjusted and stabilised, the substrates to be coated (one side polished Si wafers, glass slides and glass slides pre-deposited with a gold layer) were introduced into the deposition chamber. Finally, once the mass of the deposited plasma polymer measured using the QCM reached the pre-set value, the substrates were retracted from the deposition chamber. The thickness of all coatings investigated by XPS in this study was kept constant and as close as possible to 100 nm (see Supplementary Material Figure S1) that corresponded to the deposition time of approximately 20 min. For the NMR and gel permeation chromatography (GPC) analyses, the deposition time was 100 min, and the area of these samples deposited on a glass plate was 400 cm^2^.

### 2.2. Characterisation of Deposited Films

The chemical structure of produced samples was investigated by XPS and NMR spectroscopy.

The XPS spectra of the coatings deposited onto Si wafers were acquired by Phoibos 100 (Specs) spectrometer equipped with a hemispherical analyser (Phoibos 100, Specs, Berlin, Germany) at constant take-off angle of 90 degrees using Al Kα X-rays source (1486.6 eV, 200 W, Specs). While the survey spectra were recorded at the pass energy of 40 eV, the high-resolution XPS spectra of C 1s and O 1s peaks were acquired at pass energy of 10 eV. Before the analysis, which was performed by Casa XPS software (v.2.3.16, Casa Software, Teignmouth, UK), the XPS spectra were charge referenced for aliphatic carbon at the binding energy of 285 eV.

High-resolution ^1^H NMR spectra were recorded with a Avance 500 (Bruker BioSpin, Billerica, MA, USA), spectrometer operating at 500.1 MHz. Typical measurement conditions were as follows: π/2 pulse width 10 µs, relaxation delay 3 s, spectral width 12.5 kHz, 8 scans. The integrated intensities of ^1^H NMR signals were determined with the spectrometer integration software. ^13^C spectra were accumulated usually with 10,000 scans, with relaxation delay 5 s and spectral width 30 kHz under full proton decoupling. A ^13^C pulse duration of 8 µs was applied for the single π/2 pulse used for each scan.

Distortionless enhancement by polarization transfer (DEPT) NMR experiments were used to distinguish between CH and CH_2_ as well as CH_3_ and non-protonated carbon signals depending on the polarisation inversion time. DEPT spectra were recorded with proton decoupling and with acquisition parameters as used for ^13^C spectra. Heteronuclear single quantum coherence (HSQC) experiments were also acquired to correlate carbon atoms with their directly attached proton [36].

All NMR experiments were measured on PLA-like plasma polymer samples dissolved in deuterated chloroform CDCl_3_ at room temperature and stirred for 2 h. Tetramethylsilane (TMS) was used as internal standard, and the NMR chemical shifts are reported in parts per million (ppm) in the scale relative to TMS peak (0.0 ppm). The solubility of the samples in chloroform was estimated using the procedure analogous to the procedure described in [25].

As a supplementary technique, GPC was used (see Supplementary Material Figure S2).

## 3. Results

### 3.1. X-ray Photoelectron Spectroscopy (XPS) Characterisation of Plasma Polymerised PLA Coatings

**Figure 2:**
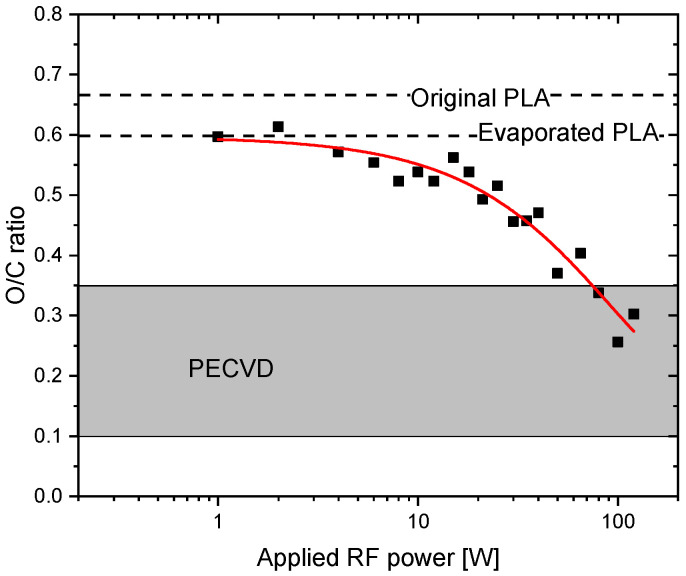
Variation of the O/C ratio in the PLA-lie films as a function of plasma power. O/C ratio of original PLA, PLA evaporated with plasma switched off (0 W) and range of O/C ratios reachable by the PECVD technique according to [31,33] are provided for comparison.

In order to understand the influence of applied RF plasma power on the chemical structure of PLA-like plasma polymer films, their chemical composition was determined using XPS, first. It was found that the films deposited at a constant deposition rate (~5 nm/min in this study) but various discharge powers significantly differ in their composition. As depicted in Figure 2, the elemental composition of the fabricated coatings at the low applied discharge power (O/C ratio = 0.6) is only slightly different from the original PLA (O/C ratio 0.66). This difference may be ascribed to the thermal degradation of PLA or to the loss of small volatile oxygen-containing molecules formed during the PLA evaporation, which are pumped out of the reactor (e.g., carbon monoxide) and do not participate in the film growth. This is consistent with the finding that the O/C ratio is similar to that one measured on the evaporated samples, i.e., samples produced with RF plasma switched off. Further increase of the applied RF power above 2 W subsequently leads to the gradually increasing carbonisation of produced PLA-like coatings that is evidenced from an almost linear decrease of O/C ratio with the plasma power (consistent with a previous study on PAVTD of PLA [25]). This behaviour is in agreement with the results obtained during the deposition of polyethylene glycol (PEO) precursor with the same technique [19]. Despite the progressively increasing fraction of carbon in the structure of prepared coatings with increasing plasma power, it is important to stress that the O/C ratio stays for the intermediate plasma powers up to 60 W considerably higher as compared to the values reported previously for the PLA-like films deposited by PECVD using ethyl lactate as a precursor [31,33]. This finding clearly proofs the differences between PECVD and PAVTD deposition techniques.

**Figure 3:**
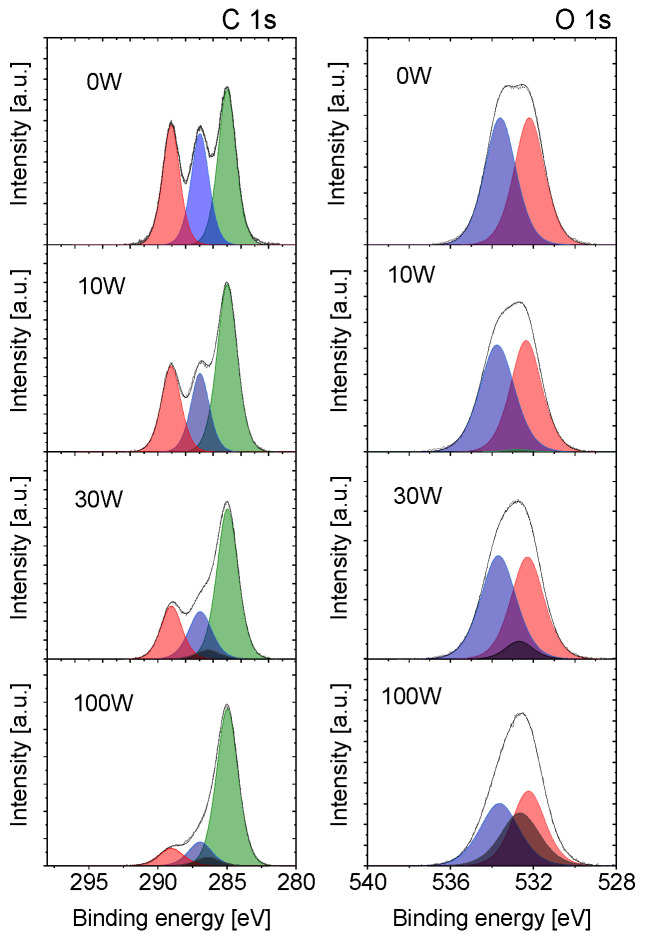
High-resolution spectra of C 1s and O 1s peaks for evaporated PLA (0 W) and PLA-like films deposited at 10 W, 30 W and 100 W (normalised). Red: C=O (C2/O2 in Figure 1b); Blue: O–(C=O)–C (C3/O1 in Figure 1b); Green: C–C (C1 in Figure 1b); Black: C–O–C ether (not present in original PLA). For details of the fitting, see Supplementary Information Figure S3.

The aforementioned alteration in the elemental composition is naturally accompanied by changes in the chemical structure of deposited thin films. This can be seen in Figure 3, where the evolutions of the C 1s and O 1s XPS peaks with discharge power are presented. The ordinary PLA XPS C1s peak can be fitted by 3 components and O1s peak by 2 components [37]. However, it was impossible to use such a simple model due to the modification of the chemical structure of PLA during the plasma polymerisation process. It is well known that plasma polymerisation leads to the destruction of chemical chains and the formation of new chemical groups. Therefore, we needed to apply a more complex model for fitting XPS spectra, including four components for C1s and two components for O1s peak. For a detailed description of the fitting procedure, see Supplementary Material Figure S3. The results may be summarised as follows.

First, for the evaporated samples with no plasma and samples prepared at low discharge powers, the C1s and O1s peaks may be deconvoluted into three components that correspond to the original PLA structure. In the case of C 1s peak, these components match with C–C/C–H bonds at the binding energy of 285.0 eV that are in PLA present in the methyl group (C1 in Figure 1b), the carbon in the carboxylic O–C=O group at the binding energy of 289.0 eV (C2 in Figure 1b) and C–O carbon in the neighbouring chain of the polymer at the binding energy of 287 eV (C3 in Figure 1b) [38]. In comparison with the PLA and in agreement with the elemental composition derived from the survey spectra (see Figure 2), the fraction of carbon in oxygen-containing bonds (~55%) is somewhat lower than in the polylactide acid (66%). On the other hand, the ratio of O–C=O and C–O carbons is 1:1, that means that the ester structure of produced films is still preserved in this power range. Following that, the O 1s peak may be deconvoluted by two peaks with 1:1 ratio that both belong to the oxygen in the carboxylate ester (C–O at 533.6 eV and C=O at 532.4 eV denoted as O1 and O2 in Figure 1b, respectively). These results that indicate the preservation of PLA-character of the coatings may also be used for the rough estimation of the average length of the chains containing PLA units not affected by the evaporation or low power plasma. This value can be calculated when it is assumed that the number of carbons in the PLA methyl group is 1/2 of the number of carbons present in O–C=O and C–O groups. Under the assumption that a certain number of PLA units is preserved, the excess of aliphatic C1 carbons represents the fraction of carbon atoms that are not existing in the original PLA. Based on the results obtained, this fraction is approximately 10–20% of all carbons, i.e., only 1–2 out of 10 carbons in the deposited films are not coming from the PLA structure. Furthermore, taking into account that there are 3 carbons per PLA unit, this means that there are in average chains composed of 2–3 undisturbed PLA units in the films deposited at low discharge powers. Although this value has to be taken only as a very rough estimate, it agrees well with the results obtained by GPC. The molar mass distribution of the films showed that the films prepared at 2 W of discharge power were composed mainly of the low molar mass oligomers with a maximum at approximately 200 g/mol, i.e., the value that corresponds to the chains composed of 3 repeating PLA units (see Supplementary Material Figure S2).

**Figure 4:**
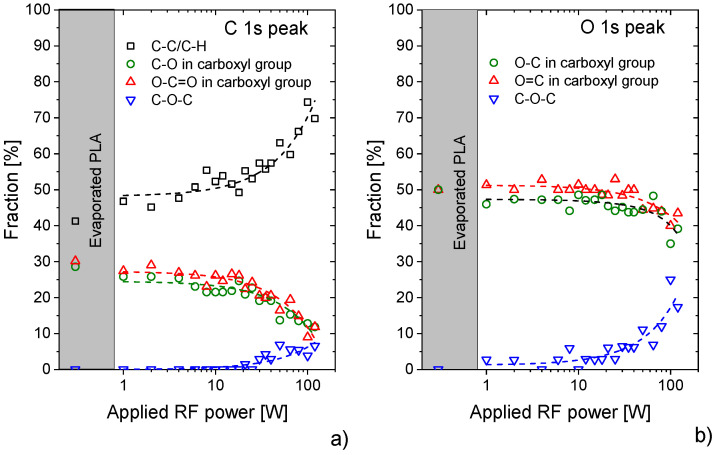
Dependence of the fraction of (**a**) carbon- and (**b**) oxygen-containing functional groups on discharge power. All corresponding XPS spectra of C 1s and O 1s XPS peaks are provided as Supporting Information Figure S3.

The second feature visible in Figure 3 is a substantial change in the structure of both C 1s and O 1s peaks with increasing the discharge power above ca. 30 W. According to the analysis of measured XPS peaks, new components had to be added to fit the spectra at the binding energies of 286.5 eV in the case of C 1s peak and 532.8 eV in the case of the O 1 s peak. These two new components correspond to C–O–C ether bonds, and their contributions gradually increase with the applied power. In addition to this, a substantial increase in the fraction of C–C/C–H bonds was observed, which shows the formation of new hydrocarbon groups in the structure of plasma polymerised PLA. The increase of aliphatic carbons and ether bonds is accompanied by the decrease of the amount of carbons and oxygens in–O-C=O and C–O groups. At this point, it is worth noting that the fractions of carbons and oxygens that may be attributed to the original PLA structure, i.e., C2 and C3 carbons and O1 and O2 oxygens, decrease at the same rate (see Figure 4a,b). Although the C2 and O1 compounds were forced to be the same in the fit, to decrease the number of free variables (see Supplementary Information Figure S3), the C3, O2 and C2(O1) differs less than 10% for all plasma powers. Such similarity in the decrease of these components means that even at the higher plasma powers, the PLA character is at least partially preserved in the PAVTD polymerised PLA films. Under this assumption and taking into account that the ratio of carbons C1, C2 and C3 are in the PLA 1:1:1, it is possible from the measured number of C2 and C3 carbon atoms to determine the relative fraction of carbons present in the original PLA structure *C_PLA_* by the equation:

(1) $$C_{PLA}=3\cdot \left(C_{2}+C_{3}\right)/2$$

where *C_2_* and *C_3_* stand for the relative contributions of C2 and C3 carbons to the total number of carbons detected by XPS. Such a defined parameter may then be used as a measure of the PLA-like character of the plasma polymerised coatings; for the original PLA structure, the *C_PLA_* is 1, while it is equal to 0 for fully hydrocarbonous random structure. In our case, the value of *C_PLA_* decreases from 0.9 for film prepared without plasma and about 0.8 for low discharge powers down to 0.3 for the highest powers employed in this study, i.e., in the manner similar to the aforementioned decrease of the O/C ratio. This means that even at the highest discharge powers 1/3 of the PAVTD deposited films are composed of PLA units not destroyed by the plasma that are randomly separated by short hydrocarbon and -C–O–C- chains.

### 3.2. NMR Analysis of PAVTD-Deposited PLA-Like Coatings

**Figure 5:**
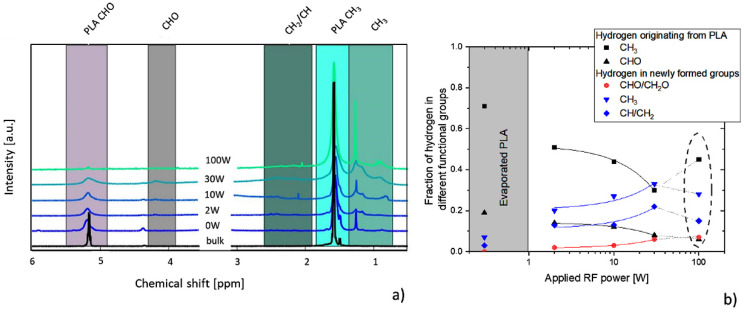
**(a**) ^1^H NMR spectra of PLA, evaporated PLA (0 W) and PLA-like coatings deposited by PAVTD of PLA at various discharge powers (**b**) changes in the composition of the films with the discharge power.

The XPS results were complemented by measurements of ^1^H NMR spectra. Unlike XPS that cannot detect hydrogen, NMR provides detailed information about different bonding states of hydrogen atoms in the samples. As can be seen in Figure 5a, in the case of bulk PLA, i.e., starting material for the PAVTD deposition of PLA-like films, two peaks were detected in the NMR spectra that belong to hydrogens in the methyl group (H1 according to the notation in Figure 1b) and to the hydrogen attached to the C3 backbone carbon of PLA (denoted as H2 in Figure 1b). In contrast, the evaporation of PLA resulted in the decrease and broadening of H1 and H2 peaks and the appearance of a peak with the chemical shift of ~1.4 ppm. This peak may be attributed to hydrogens in newly formed CH_3_ groups in the structure of evaporated PLA-like films. The switching on the RF plasma and variation of the discharge power led to further alterations in the ^1^H NMR spectra: both H1 and H2 peaks were found to decrease in their intensities with the discharge power, and new spectral peaks appeared at the chemical shifts in the regions 0.7–1.4 ppm, 1.9–2.7 ppm and 3.9–4.3 ppm. Using additional NMR experiments DEPT and HSQC performed on the sample prepared at the discharge power of 10 W (see Supporting information Figure S4), these peaks may be unambiguously assigned to CH_3_, CH/CH_2_ and CHO/CH_2_O chemical groups, respectively. In agreement with the XPS measurements, while the integral intensities of these three peaks related to the hydrogen in hydrocarbon or CHO/CH_2_O groups increased almost linearly with the increasing discharge power up to 60 W, the integral intensities of hydrogen peaks corresponding to the chemical groups present in the PLA structure decreased with power (see Figure 5b). This trend was, however, violated for the discharge power of 100 W (region marked by a dashed ellipse in Figure 5b). This looks like a controversy with findings of the XPS measurements at first sight. But unlike XPS analysis, the ^1^H NMR spectra were recorded in the liquid state, i.e., only the soluble fraction of PLA-like films was detected. The soluble fraction was greater than 80% for the films prepared at the discharge power up to 30 W, but only about 10% of the film prepared at the power of 100 W was soluble, similarly to the observed solubility of the films in water [25]. Because of this, the liquid state high-resolution NMR is not capable of fully capturing the structure of more crosslinked films deposited at 100 W.

## 4. Discussion

As was shown in the previous section, the chemical structure of PAVTD deposited PLA-like plasma polymers is strongly dependent on the applied discharge power. Both XPS and NMR, despite their intrinsically different principles, clearly showed two simultaneously occurring changes in the films with the increasing RF plasma power: (i) the increase of the hydrocarbon content that is according to NMR measurements primarily connected with the increase of the fraction of methyl groups and incorporation of new CH/CH_2_ chemical groups, and (ii) gradual rise of ether C–O bonds.

**Figure 6:**
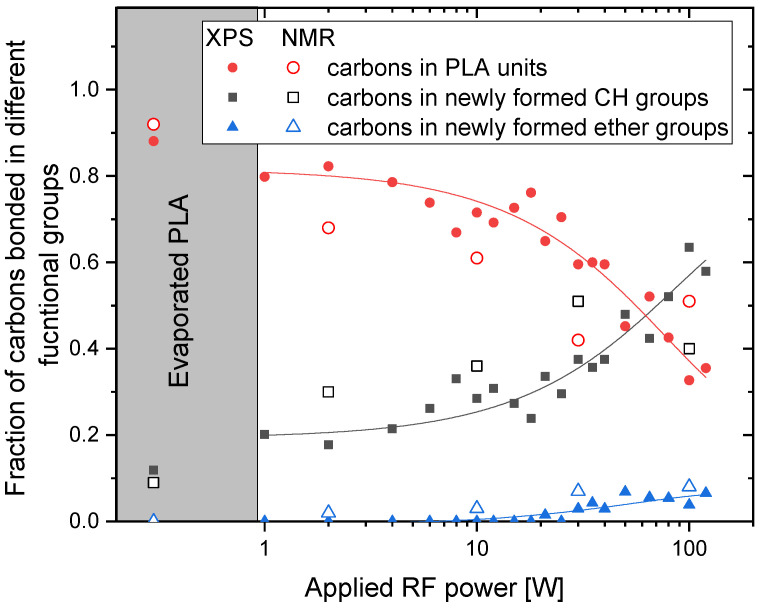
The fraction of carbon atoms bonded in different chemical groups in dependence on the discharge power as measured by XPS and NMR.

With a general knowledge about the character of the material, the fraction of carbon bonded in various functional groups calculated from XPS and NMR data were quantitatively compared. In the case of NMR, the fractions of carbons were recalculated from the measured fractions of hydrogen in different chemical groups. This recalculation was done by division of the integral for CH_3_ peaks by a factor of 3. The CH_2_/CH and CH_2_O/CHO integrals (that do not correspond to the CHO peak in the PLA structure) must be divided by a factor in the range of 1 and 2. As we observed that there are more CH2(O) than CH(O) structures, we divided the CH_2_O/CHO and CH_2_/CH by factor 2. Finally, since in the case of CHO peak originating from PLA structure there is the same amount of hydrogen as carbon, the factor used for hydrogen to carbon recalculation is equal to 1. Furthermore, the evolutions of the fraction of carbons in the ester structure of PLA and fractions of carbons in newly formed hydrocarbon and ether bonds determined by XPS and NMR gave also similar quantitative results for applied discharge powers up to 60 W (see Figure 6). As already mentioned, the significant difference that was observed for 100 W may be ascribed to the fact, that the NMR measurements were performed in the liquid state, i.e., solely on the part of PLA-like films dissolved in CDCl_3._ Besides, both employed characterisation techniques proved that especially at lower plasma powers, the original PLA ester structure was, at least partially, preserved. This is consistent with a previous study [25] where the PLA groups were confirmed by chemical hydrolysis in the films prepared both at low- and high-discharge power.

**Figure 7:**
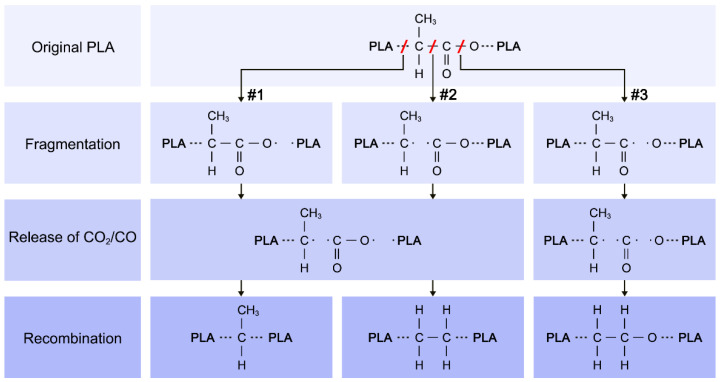
Proposed simplified reaction scheme of plasma polymerisation during the PAVTD deposition of PLA.

To explain these observations, the following simplified chemical reaction model was developed. First, taking into account the results of XPS and GPC, it is possible to suppose that the PLA is released during the vacuum thermal degradation in the form of chains of several repeating PLA units. These chains may be subsequently fragmented by the RF plasma. To keep the model simple, the fragmentations only in the backbone of PLA are considered, which in principle means that the branching of the growing plasma polymers is not allowed in the model. Although such restriction of the model is not entirely valid, it can be justified by the higher bond strength of C=O in comparison with C–C and C–O bonds and by the fact that none of the applied analytical techniques indicated a decrease in the number of methyl groups. If the PLA chain is fragmented at positions #1 or #2 according to the assignment in Figure 7, then one of the fragments formed holds either O=C*–O- or O=C–O* radical end groups as it is shown in the second row in Figure 7. Analogously, if the fragmentation of PLA occurs at the position #3, the C–C*=O is an end group of one of the created fragments. CO_2_ or CO species may be subsequently released from the created end groups. Importance of such process (especially the release of CO) was suggested by the process data on such a diverse set of experiments like magnetron sputtering of polyimide [22] or nylon [39], PAVTD of polyimide [18] or poly(ethylene oxide) [20,23]. These gases were also the most volatile species observed during purely thermal degradation of PLA [40], so these reactions shall be relevant qualitatively also to formation of the PAVTD films prepared without plasma power (0 W). The relative decrease of C=O groups in the films with plasma power was also reported for PECVD of ethyl lactate [34].

By releasing the CO/CO_2_ species, the new radicals are formed as it is schematically depicted in the third row in Figure 7. These radicals, which do not have to be a product of one fragmentation event, may merge afterwards, forming the structures presented in the last row in Figure 7. According to this reaction scheme, –CH_3_, -CH_2_–CH_2_- or –C–O–C- chemical groups are added to the structure of the resulting polymer. All of these functional groups were detected by XPS and NMR in the plasma polymerised PLA. Moreover, following the above-described schema, it is evident that the fraction of the newly formed chemical groups in the structure of deposited films increases with the number of fragmentation events. In our case, when the flux of evaporated oligomers is constant, the probability of fragmentation of evaporated PLA oligomers increases with the discharge power. This explains the observed enhanced carbonisation of the plasma polymerised PLA (reaction pathways #1 and #2 in Figure 7) as well as the increase in the number of detected ether bonds (reaction pathway #3 in Figure 7). Furthermore, the reaction scheme is also consistent with the observed reduction of oxygen concentration in PLA-like plasma polymers.

**Figure 8:**
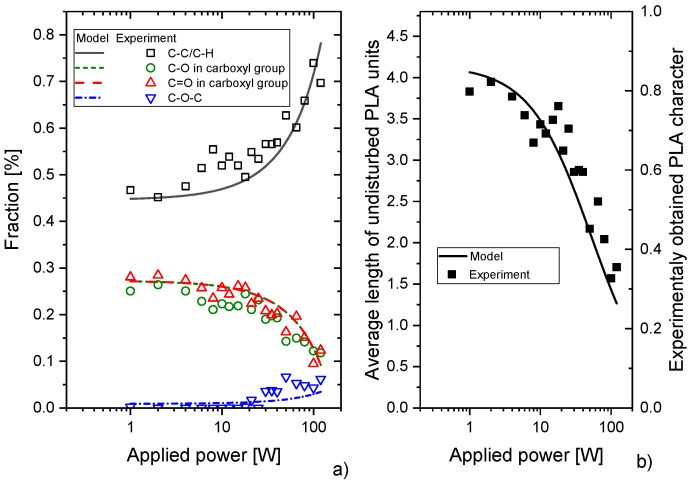
(**a**) Comparison of a model of PAVTD of PLA with experimental data. (**b**) Comparison of the evolution of the average length of undisturbed PLA units with discharge power and experimentally obtained data according to Equation (1).

To test the validity of this reaction scheme that describes the PAVTD polymerisation of PLA, the evolution of the fraction of different functional groups detectable by XPS and NMR presented in Figure 6 was modelled under the following assumptions: (i) the energy needed for the fragmentation events #1, #2 and #3 of PLA is roughly equal, and (ii) scales in the same way with the applied discharge power (in arbitrary units). These assumptions may be justified by the close values of C–C and C–O bond energies. In addition, as the rate constants of chemical pathways initiated by the fragmentation events #1, #2 and #3 of PLA are not known, their ratio may be used as a free parameter in the model. However, as the experimentally measured ratio of non-PLA C1 carbon and carbon bonded in ether groups was for all discharge powers close to ~9, the sum of processes #1 and #2 that both lead to the release of CO_2_ was set to be 9 times higher as the process #3, which causes the release of CO molecule. The results of the model are presented in Figure 8a and rescaled to be compared with the experimentally obtained fractions of carbon in different bonding states (for details, see Supplementary Material Methodology S5). Despite the aforementioned simplifications, the model describes the measured dependencies in the entire power range sufficiently. This is an interesting finding, as it shows that PAVTD deposition of PLA may be described reasonably well by a limited set of chemical reactions that is not a common situation for plasma polymerisation using volatile low-molar-mass precursors. Furthermore, the model allowed also the determination of the average length of undisturbed PLA units as a function of applied power. As depicted in Figure 8b, the average length of PLA chains decreases from about 4 in the case of low discharge powers to the value close to 1 for the power of 120 W. That corresponds to the GPC data (see Supplementary Material Figure S2) as well as to the PLA-bound carbon fraction calculated from the XPS spectra according to equation 1 (Figure 4a). The final structure of PLA-like coatings can be thus very roughly described as a sequence of individual functional units, PLA, hydrocarbon and –C–O–C- in our case. This means that the resulting material bears similarities with conventional block-copolymers or, more likely—since the side-chain reactions have been neglected in the model—as a block copolymer-like network. Despite such similarity, it is, however, worth noting, that the positions of individual building blocks are (as compared to materials produced by chemical synthesis) fully random and not predictable. Because of this, the plasma polymerisation during the plasma-assisted vacuum thermal deposition may be seen as a process that bridges the gap between a fully random character of plasma polymers and ordered conventional polymers.

## 5. Conclusions

The process of the plasma-assisted vacuum thermal deposition of polylactic acid was investigated. The study was focused on the evaluation of the influence of the discharge power on the chemical structure of resulting plasma polymer coatings. As was demonstrated, this plasma-based deposition technique represents a highly interesting alternative to the PECVD since it can produce PLA-like coatings with the chemical structure not attainable by the conventional PECVD that uses small volatile precursors. According to the presented results, it is possible just by the variation of the discharge power to synthesise either PLA-like thin films, whose structure is similar to the one of the PECVD deposited films using low-molar-mass precursors (high plasma power conditions), or films that contain several intact PLA units (low plasma power conditions), i.e., materials that resemble the structure of the original PLA. The latter pushes clearly the limits of the material obtainable using plasma polymerisation towards those prepared by the conventional chemical synthesis.

The second important finding is that both the experimental results and results of the proposed model of plasma polymerisation during the PAVTD deposition revealed that a certain number of PLA units is preserved in the fabricated samples in the whole range of discharge powers employed in this study. With increasing RF power delivered to the excitation electrode, the length of the chains PLA units decreases and they are (statistically) separated with the increasing number of either CH_3_/CH_2_ or -C–O–C- functional groups. The structure of deposited PLA-like plasma polymer coatings thus may be described in a way similar to the block-copolymers (polymer network) composed of PLA, hydrocarbon and poly(ethylene oxide)-like (ether) units. Although the position of individual building blocks in the resulting structure of produced PLA-like films is due to the stochastics nature of plasma polymerisation fully random and in contrast to block-copolymers produced by methods of chemical synthesis that are not predicable, the PAVTD technique seems to enable to control their average fraction solely by the applied discharge power. This, in turn, paves the way for the fabrication of functional PLA-like coatings with tailor-made chemical structures that can be of high interest especially in the biomedical field, e.g., for the production of thin films with well-controlled degradability, swelling or dissolving rates. Moreover, the plasma-based character of the PAVTD technique has many advantages, of which the ability to prepare compact films with a well-defined and controlled thickness on basically any substrate material is the most important, when it comes to the possible applications.

## Data Availability

The data presented in this study are available on request from the corresponding author after obtaining permission of authorized person.

## Supplementary Materials

The following are available online at https://www.mdpi.com/1996-1944/14/2/459/s1. Figure S1: Thicknesses of PLA-like coatings deposited at different plasma powers, Figure S2: The distribution of the molar masses of soluble part of PLA and PLA-like films deposited at the discharge power of 2 W measured by the gel permeation chromatography (GPC), Figure S3: (a) Measured and (b) deconvoluted C1 s and O1 s peaks at different applied discharge powers, Figure S4: NMR hydrogen, carbon, depending on the polarisation inversion time (DEPT) carbon and Heteronuclear Multiple Quantum Coherence (HMQC) spectra of the sample prepared at 10 W of plasma power used for the assignment of peaks in ^1^H NMR spectra, Methodology S5: The calculation of the composition of the PAVTD films according to the model.
