# Supplementary material for: Structure of Plasma (re)Polymerized Polylactic Acid Films Fabricated by Plasma-Assisted Vapour Thermal Deposition
Source: Materials (Basel). 2021 Jan 19;14(2):459. doi: 10.3390/ma14020459 (PMC7832887; doi:10.3390/ma14020459)
Supplement: Supplementary file 1 [file materials-14-00459-s001.pdf]

materials

Supplementary Information

Supplementary Materials: Structure of plasma (re)polymerized polylactic acid films fabricated by plasma-assisted vapour thermal deposition

Zdeněk Krtouš, Lenka Hanyková, Ivan Krakovský, Daniil Nikitin, Pavel Pleskunov, Ondřej Kylián, Jana Sedlaříková and Jaroslav Kousal

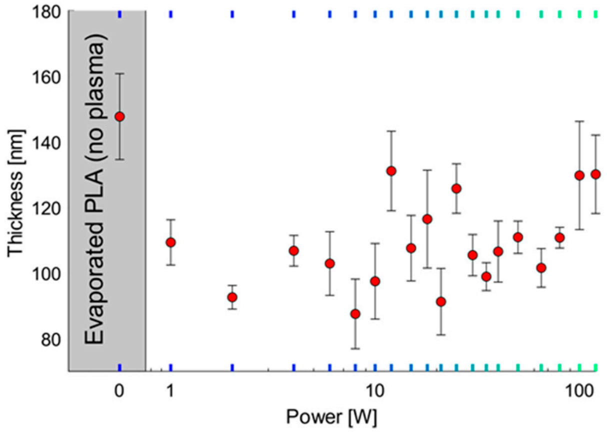

Figure S1. Thicknesses of PLA-like coatings deposited at different plasma powers. The thickness of the films was determined by Woollam M-2000DI spectroscopic ellipsometer. The measurements were done at three angles from 50° up to 70° and in the wavelength range of 192 nm–1690 nm in the air at room temperature. The ellipsometry data were fitted using a CompleteEASE software (J.A. Woollam) according to Cauchy model for silicon substrate with 1.5 nm of native oxide.

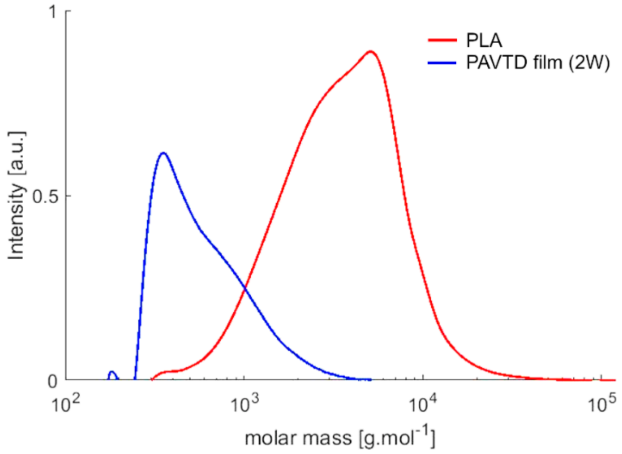

Figure S2. The distribution of the molar masses of soluble part of PLA (red) and PLA-like films deposited at the discharge power of 2W (blue) measured by GPC. GPC measurements were performed using the Agilent 1260 Infinity GPC/SEC system equipped with a differential refractometric detector. The separation system consisted of a 7.5 × 600 mm Polymer Laboratories gel column of porosity 1000 Å, and it was calibrated with polystyrene standards. Tetrahydrofuran (THF) was used as a mobile phase with a flow rate of 0.8 mL·min^{-1} at temperature 30 °C. For purposes of chromatography, ca 3% (w/v) solutions of the PLA and PLA-like plasma polymer deposited at the discharge power of 2W in THF were prepared by intensive stirring using an electromagnetic stirrer at room temperature. Insoluble (gelled) part of the material (<25%) was filtered off using a fine 0.2 µm Teflon membrane filter.

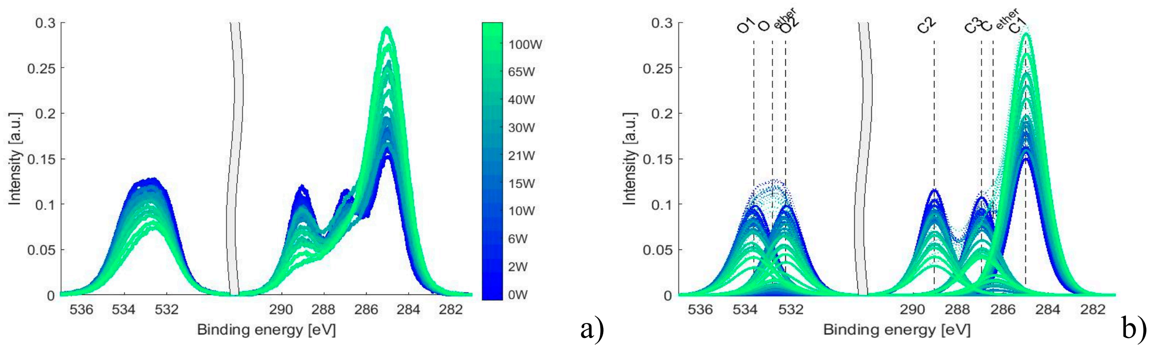

Figure S3. (**a**) Measured and (**b**) deconvoluted C1 s and O1 s peaks at different applied discharge powers. The colour gradient distinguishes between the samples varying in the power of plasma used for their preparation. In the case of peak fitting, the areas of peaks that correspond to carbon and oxygen bonded in O–C=O carboxyl group were set to be equal.

Commentary: In this section, we want to justify the presence of the 7-component fit. Although it is possible to fit the data just with the C1, C2, C3 for C1s and O1, O2 for O1s components belonging to the original PLA, we found that the discrepancies between the measured data and the model increase with the discharge power. However, these discrepancies alone would not justify adding another component.

In the 5-component model, it was found that the C3(C–O) and O2(O=C) components (with consideration of the relative sensitivity factor) have almost the same contribution for all powers. On the other hand, the C2(C=O) and O1(C–O) also have a similar contribution for all powers, which differs from the two previously mentioned. Such a trend was found unlogic and suspicious. Correlations between the C2(C=O) and O2(O=C) and also between C3(C–O) and O1(O–C) shall be present.

This issue was possible to fix by adding C_{ether} and O_{ether} components. With this fix the C2, C3, O1 and O2 contribution ratios are almost the same for all powers. The difference in binding energy between C3 and C_{ether} peak is approximately 0.5 eV, barely sufficient to justify the addition of another component. Moreover, such an approach can be easily seen as overparameterization of the fit of O1s peak. Therefore, the number of free parameters was decreased by fixing the energies of all peaks according to the XPS database. Binding energies for C_{ether} and O_{ether} were taken from tabulated values for PEG polymer as it is a natural example of an ether-like polymer. Also, the O1(O=C) contributions were set to be equal to the contribution of C2(C=O) with respect to the relative sensitivity factor. Then the O1s component is fitted with just two components (O2, O_{ether}); the third component is fixed. With such an approach, the chemical interpretation seems to match other results (NMR).

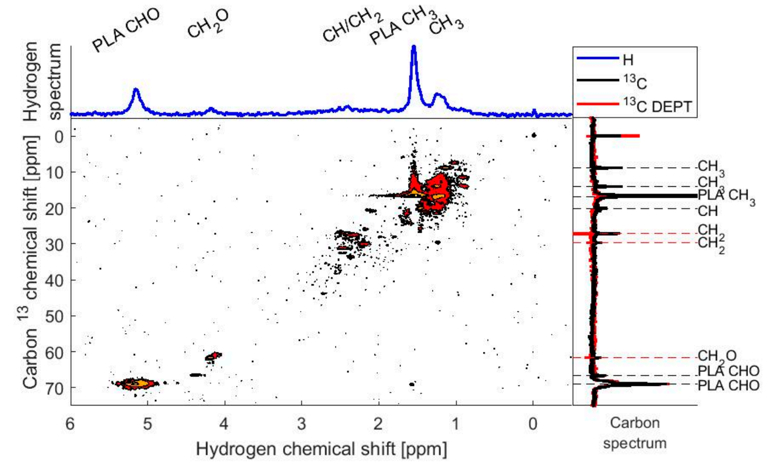

Figure S4. NMR hydrogen (blue), carbon (black), DEPT carbon (red) and Heteronuclear Multiple Quantum Coherence (HMQC) spectra of the sample prepared at 10 W of plasma power used for the assignment of peaks in ^{1}H NMR spectra.

Methodology S5.

The calculation of the composition of the PAVTD films according to the model discussed in the main text of the paper was done as follows.

Based on the reaction scheme in Figure 7, it is possible to evaluate the change of the number of elements in the polymer chain after one reaction. These changes are summarized in the table:

	C1	C2	C3	O1	O2	C_{ether}	O_{ether}
$\Delta X_{\#1}$	1	-1	-1	-1	-1	0	0
$\Delta X_{\#2}$	1	-1	-1	-1	-1	0	0
$\Delta X_{\#3}$	1	-1	-1	-1	-1	1	1

Therefore, the number of element X^N atoms in the chain after N reaction can be expressed as

$$X^N = X^0 - N\,(\alpha_{\#1}\,\Delta X_{\#1} + \alpha_{\#2}\,\Delta X_{\#2} + \alpha_{\#3}\,\Delta X_{\#3}) \quad \text{(S1)}$$

where X^0 is the number of corresponding elements in bulk PLA and $\alpha_{\#i}$ are the relative rate constants of the reactions. As discussed in the main text, this was set as: $\alpha_{\#1} + \alpha_{\#2} = 0.9$, $\alpha_{\#3} = 0.1$

When the initial polymer chain is assumed to be sufficiently long ($X^0 >> 1$), the composition of the film after N reactions can be calculated. The number of reactions that the polymer chain experiences during the process is assumed (in case of the constant evaporation rate) to increase linearly with the discharge power. The scaling of the composition of the films predicted by the model to real discharge powers was done as follows.

Substantial changes in the polymer chain occur already during the thermal degradation of the polymer in the crucible when the fragments that subsequently enter the plasma are formed. The plasma (re)polymerization shall add more reactions, proportionally with the discharge power. Since the thermal processes cannot be compared to the plasma processes directly, we match the experimental data on the resulting film composition in two points, at low and high plasma power. Experimental data on the amount of carbons denoted as C1 for low power (1W) and high power (80W) were used to find N values that lead to same values of C1 atoms amount in the model.

$$C_1^{[1W]} = C_1^{[N_1]} \quad \text{(S2)}$$

$$C_1^{[80W]} = C_1^{[N_2]} \quad \text{(S3)}$$

With the assumption that the number of reaction is linearly dependent on the number of active species in plasma and this is linearly dependent on the plasma power, the scaling factor between the number of reactions and the plasma power can be found:

$$P[W] = N\,\frac{80[W] - 1[W]}{N_2 - N_1} \quad \text{(S4)}$$

The outcome of these equations is summarized in Figure 8 of the main text. While the absolute scaling of N(P) cannot be obtained in this way, the authors deem the possibility to describe the composition of the plasma polymer by such a simple model as remarkable per se.
